# Application of repeat image analysis to radiation therapy imaging modalities as a quality improvement tool for image guided radiotherapy

**DOI:** 10.1002/acm2.14019

**Published:** 2023-05-04

**Authors:** Daniella Chee, Lesley Buckley

**Affiliations:** ^1^ Radiation Therapy Department Princess Margaret Hospital Toronto Ontario Canada; ^2^ Medical Physics Department The Ottawa Hospital Ottawa Ontario Canada; ^3^ Department of Radiology, Radiation Oncology and Medical Physics University of Ottawa Ottawa Ontario Canada

**Keywords:** IGRT, radiation therapy, repeat image analysis

## Abstract

**Background:**

Repeat images contribute to excess patient dose and workflow inefficiencies and can be analyzed to identify potential areas for improvement within a program. Although routinely used in diagnostic imaging, repeat image analysis is not widely used in radiation therapy imaging, despite the role of imaging in the delivery of precise radiation treatments.

**Purpose:**

Repeat image analysis was performed for on‐board cone beam CT imagers and CT simulators within a radiation therapy department. Both the rate of repeat images and the reasons for the repeat images were analyzed.

**Methods:**

Data from nine conventional linear accelerators and three CT simulators were analyzed retrospectively over a 5‐month period. Repeated images that were not part of the standard of care were considered repeat images. The repeat rate was expressed as the number of repeat scans as percentage of the total number of scans performed. The reasons for the repeats were collected and classified as either patient preparation, patient setup, patient motion, or machine error. These reasons were further classified into sub‐categories.

**Results:**

The overall repeat rate across the linear accelerators was 3.3%, with a maximum of 5% on any single machine. The repeat rate for the three CT simulators was 1.5%. The most common reasons for repeat images were patient preparation (incorrect bladder or rectal filling) and patient setup or positioning. Greater positioning challenges led to higher repeat rates on units that treat a large number of breast patients, palliative patients, or pediatric patients.

**Conclusions:**

Repeat image analysis can be applied within a radiation therapy department. Establishing baseline repeat rates and analyzing reasons for the repeat images can identify opportunities for improvements in terms of patient dose reduction and workflow efficiency for the program. Periodic repeat image analysis also permits monitoring the program for changes and for comparison against rates at other institutions.

## 1 INTRODUCTION

Repeat image analysis is a key component of a quality assurance program in diagnostic imaging,^1^ is mandatory for mammography installations^2^ and is recommended by many organizations for other diagnostic facilities.^1^, ^3^, ^4^ Repeated images contribute to excess patient dose and workflow inefficiencies. Identifying repeat rates and the reasons for repeated images helps identify potential issues with machine performance, staff training, and clinical protocols and monitors an imaging program for drift in practice over time.^5^

Repeat image analysis involves the review of all repeated imaging scans in order to identify reasons for the rescans and patterns in the data.^6^ Patient scans that are repeated for any reason are recorded, along with the reason for the rescan. These images can be further categorized in terms of clinical procedure, imaging unit, staff performing the scan, and any other criteria that may help identify factors contributing to the need for repeat imaging. A common parameter considered during repeat image analysis is the repeat rate which indicates the percentage of scans that require a repeat scan.

Several published guidance documents and studies provide recommended and typical values of repeat rates for a diagnostic imaging department.^1^, ^3^, ^7^ These guidance values provide a baseline against which an imaging department can evaluate their own repeat rates. Repeat rates above or below typical values may indicate problems within the imaging practice that could be addressed. The aim of repeat image analysis is not to eliminate all repeat images since unpredictable patient motion, machine errors and human errors on the part of the staff are unavoidable. However, repeat image analysis can identify outliers in terms of the imaging unit, staff member or the institution itself relative to recommended values. Identifying outliers allows for system improvements to reduce the rate of avoidable repeat images.

There is a lack of published data on repeat image analysis in radiation therapy. Imaging plays an important role in radiation therapy treatments, providing verification of the correct patient position and the location of internal anatomy that cannot be assessed externally. This permits the use of highly conformal dose distributions which offer the potential to deliver higher doses to target volumes while sparing normal tissues. Imaging increases the integral dose to the patient and though the dose from imaging is small compared to the therapeutic dose,^8^ it is not negligible and the aim of all clinical imaging should be to keep doses as low as reasonable without compromising image quality.

The intent of performing repeat image analysis within a radiation therapy department is to assess the frequency and reasons for repeat images on the CT simulation units and on the treatment units. By identifying reasons for repeat images, the program can assess whether some of these repeat scans could be eliminated through changes to clinical processes. Imaging and image registration account for a significant portion of the total treatment time in radiation therapy, so a reduction in the rate of repeat imaging can improve patient throughput, treatment time for the patient, and overall scheduling efficiency for the program.

In the absence of published data for radiation therapy imaging, performing an initial repeat image analysis will serve as a baseline for subsequent reviews. Further, understanding the frequency of repeat imaging gives information on the clinical workload and can be used to identify potential improvements within the system.

## 2 METHODS

The study considered data from nine linear accelerators equipped with kilovoltage cone beam CT (CBCT) systems and from three CT simulators dedicated for use in radiation treatment planning within a single radiotherapy department. All linear accelerators are from the same vendor and are matched in terms of photon beams. Two have electron capability and all have the same on‐board imaging. Three of the machines are equipped with a 6‐degree of freedom couch used to correct patient rotations during setup. The machines are matched so that patients can be transferred between units and staff are able to move from one unit to another. For the efficiency of clinical workflow, patients with similar disease sites and treatment techniques are grouped together in specific units. The three CT simulators are in every way identical to one another. The nine accelerators and three simulators are spread across two geographic sites, with three linacs and one simulator at one site and all of the other machines at the other site. Most staff are assigned to one site only, however, there are some staff who move between the two locations.

For the purposes of the data collection, all CT or CBCT scans performed during a 5‐month period were reviewed retrospectively, and any scans that had been repeated were identified. Repeat scans were identified from the electronic patient records in the record and verify system (MOSAIQ, Elekta Inc.). For the CT simulation scans, repeat scans were identified by flagging any patient for whom there were multiple scans on a single day. Patients for whom multiple scans were prescribed as part of the standard of care (e.g., full and empty bladder scans) were not considered to be repeat images. For unplanned repeat images, outside the standard of care, the reason for the repeat was normally entered into the appointment information. For the CBCT scans, repeat scans were captured as part of the workload capture for the patient, but there were inconsistencies in where the reason for the additional scan was recorded.

Patients who received only other imaging modalities, such as 2D portal imaging, were not included in the analysis. Over 90% of patients at the center receive a daily CBCT for positioning verification, meaning that the greatest gains in efficiency from potential process improvements lie with the CBCT process. Other imaging modalities such as 2D kV imaging or MV portal imaging are either not used (2D kV imaging) or used very seldom at our center (portal imaging). All patients who received either a CBCT or CT simulation scan were included, regardless of age, disease site, or other factors.

The total number of scans conducted on each unit during the time period considered was recorded, as was the number of repeat images. The repeat rate was computed as the ratio of repeat images to a total number of scans and was computed for each unit individually, as well as for all units in aggregate.

Repeat images were classified in terms of the reason for the repeat scan and a category was included for any patients where no reason for the repeat was recorded. The categories were selected to represent common reasons for repeat imaging within the radiotherapy context. These reasons are similar to the repeat reasons normally used in diagnostic imaging but were selected to represent specific issues within the radiation therapy imaging process. No patient identifiers were included with the data.

## 3 RESULTS

**FIGURE 1:**
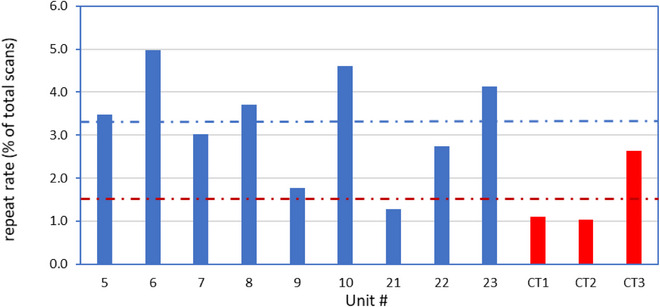
Repeat rates for the nine conventional linear accelerators (blue) and three CT simulators (red). The average repeat rates across the nine linacs and three simulators of 3.3% and 1.5% are shown as dashed lines.

Figure 1 shows the repeat rates for each of the units included in the study, as well as the overall repeat rates for both the linear accelerators and CT simulators. The overall repeat rate across the nine conventional linear accelerators for the 5‐month period was 3.3%. There was some variation between individual treatment units, with the highest repeat rate for a single unit being 5%. There was no noticeable difference between the repeat rates at the two different geographic sites. (The treatment units with in‐house designations 21, 22, and 23 are located at the separate site from the other treatment units.) The repeat rate was lower on the CT simulators, with an average value of 1.5% for the three simulators. The two simulators at the same geographic location (in‐house designation CT1 and CT2) showed a similar repeat rate to each other, whereas the repeat rate was higher for the third simulator which is located at a separate site.

The reasons for the repeat images were categorized into four broad categories based on the most common reasons indicated in the patient record. These categories were patient preparation, patient motion, patient setup, and machine error. Repeat images for which no reason was specified were grouped separately. Patient preparation included rescans when the patient anatomy in the initial image did not conform to pre‐treatment instructions (e.g., insufficient bladder filling). The patient setup included other issues with the patient positioning that were not related to patient preparation instructions. The patient motion refers to scans that had to be repeated because the patient moved during the image or prior to treatment and the machine errors included any machine interlock that required a re‐scan. Each category was further classified into sub‐categories in order to distinguish between causes of the repeat imaging The machine‐related errors included a separate category for errors related specifically to the 6 degree of freedom couch system since these appeared frequently on the three units that are equipped with this system. Table 1 summarizes the reasons for repeat images used in the study.

**TABLE 1:** Categories and sub‐classifications of reasons for repeat imaging on the linear accelerators or CT simulators.

Category	Sub‐classification
Patient preparation	Incorrect bladder filling
	Incorrect rectal filling
Patient motion	Changes to internal anatomy
	External patient motion
Patient setup	General positioning error
	Rotations
	Arm/shoulder position
	Chin/head/neck position
	Machine clearance
	Accessory adjustment
	Unclear instructions
Machine error	Machine communication error
	6 degree of freedom couch error
	Other
No reason specified	

**FIGURE 2:**
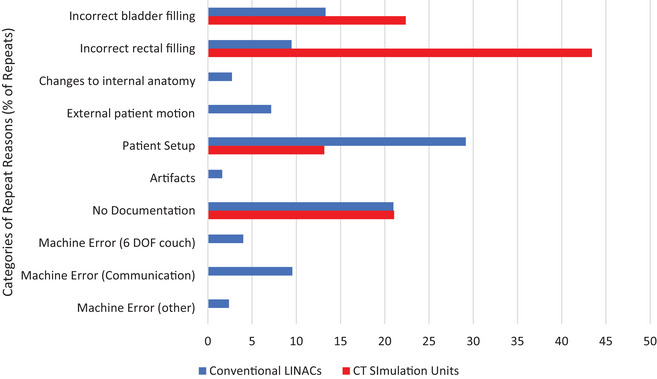
Frequencies of repeat reasons, presented as a percentage of the total number of repeat images. The data for the CT simulation units is shown in red, and for the linear accelerators in blue.

On the CT simulators, the most common reason for a repeat image was patient preparation, either related to bladder or rectal filling. This accounted for nearly 68% of all repeat images. The only other category documented as a reason for a repeat CT image was patient setup. In 21% of cases, no reason for the repeat image was documented. For the CBCT images, patient setup accounted for 29% of all repeat images and was the most common reason for a repeated scan. Patient preparation was documented 23% of the time and in 21% of cases, there was no reason recorded for the repeat image. Figure 2 summarizes the frequencies of reasons for repeat imaging, as a percentage of the total number of repeat images.

**FIGURE 3:**
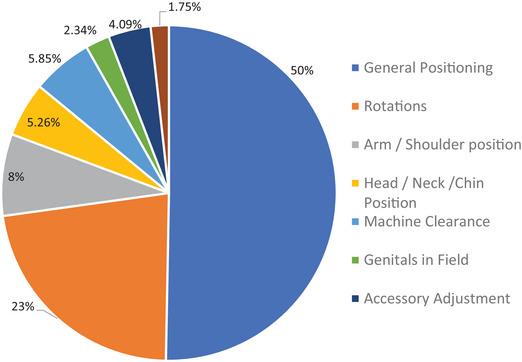
Distribution of the sub‐categories of patient setup errors leading to repeat images.

Within the patient setup category, 50% of repeat images were due to general issues with positioning, meaning that the patient positioning could not be adequately confirmed with the image registration and the patient needed to be repositioned and rescanned. Patient rotations accounted for 22% of the repeat images. Patient rotations at the time of setup can be corrected by the machine only on the three units with a 6‐degree of freedom couch. In other instances, or if the rotation is too large, the patient has to be re‐positioned and re‐imaged. Figure 3 shows the breakdown of patient setup errors leading to repeat images.

**FIGURE 4:**
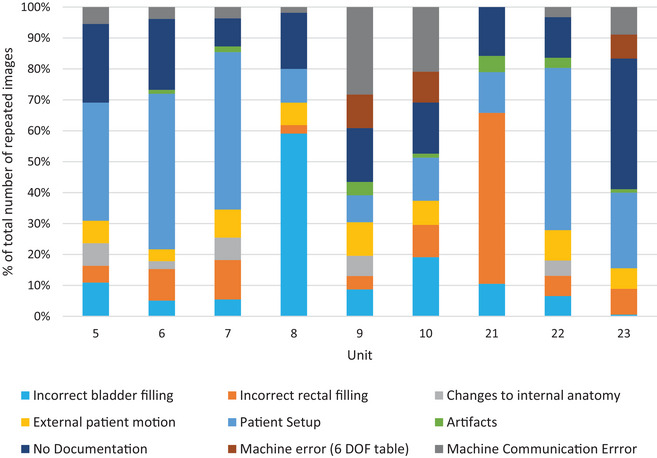
Relative frequency of repeat reasons, shown for each treatment unit.

Patients are grouped on the treatment units according to disease site when possible. This standardizes the patient setups and increases staff expertise with specific treatment sites, leading to increased efficiency in the clinical workflow. This also results in certain patient setup issues being more common on some units as seen in Figure 4 which shows the relatively frequency of reasons for repeat imaging as a function of treatment unit for the linear accelerators. Treatment units 5, 6, 7, and 22 show a higher percentage of patient setup causing a repeat CBCT. These units treat primarily breast cancer patients, palliative patients, and pediatric cases. Units 8 and 21, which treat mostly prostate cancer patients show the highest proportion of repeats caused by patient preparation issues. There were also differences observed in the units that have the 6‐degree of freedom treatment table (9, 10, 23). These three units had a higher rate of machine communication errors due to the table software, with units 9 and 10 showing a higher rate than 23.

## 4 DISCUSSION

The repeat rates seen in radiation therapy imaging are lower than those typically reported for diagnostic imaging departments.^5^, ^7^ Although there are no published data on repeat image analysis in radiotherapy, the rate of repeats is expected to be lower since the endpoint for the imaging is to verify patient positioning, rather than diagnosis. This allows a greater tolerance of minor deviations, provided that the relevant anatomy can be confirmed. There is also very robust patient positioning and immobilization in place to ensure reproducibility throughout the multiple days of treatment, so potential issues such as patient motion are reduced. At the level of the CBCT systems, the imaging is used to confirm the alignment of the patient positioning with the reference image acquired at the time of the CT simulation. As such, there are more stringent requirements on the positioning at the time of treatment than there are at the time of the CT which is used to set the baseline. It is for this reason that we expect a higher rate of repeat images on the CBCT units than on the simulators.

The reasons for repeat images are similar to those found in diagnostic imaging repeat image analysis, however in radiotherapy there is an increased emphasis on internal anatomy in order to optimize the dose delivery. This requires specific patient preparation instructions to ensure an accurate dose delivery. Patients are frequently treated with specific bladder and rectal filling requirements, leading to a relatively high rate of repeat images on the treatment units as this is not something that can be assessed until an image has been acquired. Although other patient preparation issues such as diet and respiratory rhythms could also lead to the need for repeated imaging, these were not observed in this data set. Other causes of repeat images such as patient motion, which is frequently seen in diagnostic imaging, are less common in radiation therapy since the patient positioning is carefully designed to limit variability in patient position from day to day and throughout the treatment delivery which takes significantly longer than the image acquisition.

Treatment units that treat a large number of pelvic patients will have a higher percentage of patient preparation repeat images since this is the largest patient population that has strict pre‐treatment bladder and rectal filling instructions. There is a higher percentage of patient setup issues requiring re‐imaging on units that treat primarily palliative patients or breast cancer patients. Patients undergoing palliative radiation therapy may be experiencing a significant amount of pain and positioning can be challenging. Breast radiotherapy also poses positioning challenges since breast tissue is difficult to achieve a reproducible position and the patient position itself, with arms above the head, can lead to greater variation and risk of collisions with the treatment unit. Patient setup challenges however are not limited to these two sites and can present with any patient cohort.

Repeat image analysis can sometimes be used to identify differences in machine performance or staff behaviors. The three linear accelerators that have a 6‐degree of freedom treatment table (9, 10, and 23) use an additional software program to control the table and all three had instances of repeat images due to errors within that software. The rate of repeat images due to these communication errors was not consistent across the three units, suggesting potential differences in software or machine performance, although at this time no specific cause for this difference has been identified. Differences observed between the image repeat rates between the CT simulators at the two geographic locations suggest possible differences in thresholds used by the staff at the two sites for re‐imaging. All repeat images were due to either patient preparation or patient setup, either of which requires judgment on the part of the staff performing the image. CT staff at the two sites follow the same training process and the patient workload does not differ significantly between the sites, however, most training is peer‐to‐peer so it is possible that differences in approach exist between the two staff groups at the two sites. The small number of repeat images and simulators makes further analysis difficult and does not warrant additional investigation.

A high percentage of repeat images (21%) were found to have no documented reason for the repeat image. This is a shortcoming within the patient record since there should be documentation supporting any additional scan. This, along with the observed variation in where the reasons for the repeats were documented in the patient record, points to a lack of standard procedure for the staff to follow in such cases. A standard procedure, along with building staff awareness of the importance of recording the reasons for the repeat images, are important features in building quality improvement initiatives within the radiation therapy imaging process.

## 5 CONCLUSION

This study has demonstrated that repeat image analysis, as used in diagnostic imaging, can be applied within a radiation therapy department. Although repeat rates in radiation therapy are lower, repeat images nonetheless contribute to excess patient dose and cause inefficiencies within the system. Imaging dose is a small fraction of the overall patient dose in radiation therapy, however analyzing the reasons for repeat images within a radiotherapy department can identify areas for improvement, from standardization of documentation, to training of staff across multiple sites to differences in machine performance. Repeat image analysis can play an important role in continuous quality improvement initiatives within a radiotherapy program.

Specific to this study, the relatively high rate of undocumented reasons for repeat images indicates the need for a program‐wide protocol for documenting these occurrences. Furthermore, a baseline repeat rate of 3.3% on the linear accelerators and 1.5% on the CT simulators has been established. This can be used for subsequent continuous quality improvement initiatives, including establishing a periodic review of repeat imaging rates within the department. It can also serve as a baseline against which other centers wishing to do a similar analysis can compare.

## AUTHOR CONTRIBUTIONS

Both authors (Daniella Chee and Lesley Buckley) were involved in the project design and data collection. The manuscript was drafted by L Buckley and was reviewed and edited by both Daniella Chee and Lesley Buckley. Both authors agree to its submission for publication.

## CONFLICT OF INTEREST STATEMENT

The authors have no conflicts of interest to disclose.
